# Access, awareness, and risk: drivers of unsafe sharps disposal and needle reuse in insulin-treated adults in China

**DOI:** 10.3389/fpubh.2025.1698224

**Published:** 2025-11-13

**Authors:** Shaoping Wu, Fang Wang

**Affiliations:** 1 Department of Endocrinology, The Central Hospital of Enshi Tujia and Miao Autonomous Prefecture, Enshi, Hubei, China; 2 Department of Outpatient, The Central Hospital of Enshi Tujia and Miao Autonomous Prefecture, Enshi, Hubei, China

**Keywords:** insulin injection, sharps disposal, needle reuse, community safety, take-back programs, China

## Abstract

**Background:**

Unsafe household sharps disposal and needle reuse among insulin-treated adults pose clinical and public health risks; however, population-based data from China are limited.

**Methods:**

We conducted a cross-sectional survey of consecutive insulin-treated outpatients at a tertiary hospital in Enshi, Hubei (February 2023–December 2024). A cross-culturally adapted questionnaire captured 30-day disposal and reuse practices, community-safety incidents, and access determinants. Primary outcomes were unsafe disposal (household trash, public bins, or toilets/sinks) and any needle reuse. Multivariable logistic regression estimated adjusted odds ratios (aORs) with 95% confidence intervals (CIs).

**Results:**

Among 1,810 insulin-treated adults, unsafe sharps disposal was widespread (59.0%; 95% CI: 56.7–61.2%). Disposal methods included loose trash at 43.5%, public bins at 15.9%, and approved containers at 24.7%. Needle reuse was reported by 32.5% (95% CI: 30.4–34.7%); among reusers (*n* = 588), the primary reason was cost (47.1%). In adjusted models, unsafe disposal was associated with younger age (<30 years, aOR: 2.31; 95% CI: 1.42–3.76; 30–44 years: aOR: 1.67; 95% CI: 1.28–2.18), male sex (aOR: 1.38; 95% CI: 1.14–1.67), lower education attainment (no formal/primary education: aOR: 2.84; 95% CI: 2.02–3.99), low income (aOR: 3.42; 95% CI: 2.31–5.07), travel time >30 min (aOR: 1.94; 95% CI: 1.51–2.49), lack of awareness about take-back programs (aOR: 2.12; 1.75–2.57), and limited availability of containers (aOR: 2.45; 95% CI: 2.01–2.98), pump usage (aOR: 0.45; 95% CI: 0.27–0.75), and counseling (aOR: 0.35; 95% CI: 0.29–0.43). Needle reuse showed similar patterns: younger age (aOR: 1.89; 95% CI: 1.18–3.03), male sex (aOR: 1.29; 95% CI: 1.07–1.55), lower education (aOR: 2.16; 95% CI: 1.56–2.99), low income (aOR: 2.87; 95% CI: 1.95–4.24), diabetes duration ≥10 years (aOR: 1.34; 95% CI: 1.11–1.61), travel time >30 min (aOR: 1.78; 95% CI: 1.39–2.28), lack of awareness (aOR: 1.87; 95% CI: 1.55–2.26), limited availability of containers (aOR: 3.21; 95% CI: 2.64–3.90), pump usage (aOR: 0.31; 95% CI: 0.15–0.64), and counseling (aOR: 0.42; 95% CI: 0.35–0.51). Community safety risks increased: unsafe disposal raised the odds of household needlestick (aOR: 3.47; 95% CI: 2.01–5.99) and observed the presence of needles in the community (aOR: 2.89; 95% CI: 1.85–4.52). Additionally, the reuse of needles has also increased, impacting both household needlestick incidents and community needle observations (aOR: 2.23; 95% CI: 1.42–3.50; aOR: 1.67; 95% CI: 1.13–2.47).

**Conclusion:**

Unsafe disposal and needle reuse are prevalent and exhibit strong socioeconomic gradients. Targeted education, container provision, and accessible take-back programs may improve patient and community safety in China.

## 1 Introduction

The safe handling and disposal of sharps among adults with diabetes who self-inject insulin constitutes a critical, yet often under-addressed, dimension of both clinical quality and community safety (1–3). Inappropriate practices—particularly needle reuse and household discarding of used needles and lancets—elevate risks ranging from suboptimal insulin delivery and skin complications to needlestick injuries among family members, sanitation workers, and the broader public (1, 4). Therefore, international consensus recommendations emphasize single-use needles, correct injection technique, and puncture-resistant container disposal as foundational elements of safe diabetes care outside clinical environments (5, 6). However, community safety is defined as the protection of household members, waste-collection personnel, sanitation workers, and the general public from needlestick injuries and potential blood-borne pathogen exposure resulting from improper sharps handling and disposal practices in non-clinical settings. This encompasses both direct household risks (e.g., injuries to cohabitants, especially children) and broader public risks (e.g., exposures to sanitation workers and discovery of discarded needles in public spaces) (2–5).

Despite clear guidance, needle reuse remains common in real-world settings, driven by convenience, misconceptions, and cost barriers, and multiple studies have documented its prevalence across diverse populations of insulin users (6, 7). Reuse has been linked mechanistically and observationally to injection-site lipohypertrophy, dose-delivery variability (including pen needle clogging), and increased discomfort, although short-term effects on glycemic indices appear heterogeneous across studies (8, 9). Notably, older experimental and observational reports suggesting minimal glycemic impact with limited reuse must be interpreted cautiously, considering contemporary device designs and current consensus discouraging any reuse (9, 10).

Suboptimal sharps disposal is similarly prevalent and multifactorial in origin, with community-based surveys repeatedly demonstrating that a substantial proportion of insulin users discarded used sharps in household trash rather than using approved collection systems (2, 11). In a synthesis of various settings, authors have underscored that as the prevalence of diabetes increases, the volume of household-generated sharps will rise commensurately, amplifying downstream public-health hazards without significant improvements in disposal infrastructure and education (1, 2). Evidence from outpatient cohorts further indicates that formal instruction on disposal triples the odds of correct practices, highlighting education as a modifiable determinant of safe behavior (12, 13).

The community-safety implications of improper disposal extend beyond the individual with diabetes to cohabitants, waste-collection personnel, custodial staff, and first responders (3). Occupational studies of waste handlers indicate that there are measurable rates of sharps injuries attributable to needles discarded in thin plastic sacks or overfilled containers, with attendant physical harm and psychological distress, even in cases where seroconversion does not occur (13, 14). Although the absolute risk of blood-borne virus transmission from community-acquired needlestick injuries is low, clinical guidance emphasizes careful assessment, counseling, and follow-up due to non-zero risks and considerable anxiety (15). In cases where a needlestick involves an infected source, the per-exposure transmission probabilities for Hepatitis B Virus (HBV), Hepatitis C Virus (HCV), and Human Immunodeficiency Virus (HIV) underscore the importance of primary prevention through safe handling and disposal of sharps at home (16, 17).

China represents a particularly salient context in which the triad of needle reuse, sharps disposal, and community safety converges with system-level barriers (4). Large multicenter and survey-based investigations in mainland China report high rates of needle reuse and notable burdens of lipohypertrophy, with the cost of pen needles, injection-technique skill deficits, and duration of insulin therapy frequently implicated as drivers (4, 18, 19). In addition, economic analyses indicate that the lack of reimbursement for pen needles may negatively influence technique quality and resource utilization, thereby sustaining reuse behaviors (20, 21). Nevertheless, observational work indicates persistent gaps in the consistent provision and uptake of such training across clinical settings, reinforcing the need for intervention research tailored to real-world constraints (22, 23). Recent work localizing sharps-disposal questionnaires for Chinese settings underscores structural constraints: many patients lack access to sharps containers and community collection terminals, and therefore dispose of sharps with household waste; knowledge and attitudes strongly predict disposal practices (24). A 2023 systematic review focused on sharp-waste disposal outside medical institutions further highlights the scarcity of standardized community pathways for safe collection, with attendant public-safety implications as at-home injections proliferate (4).

China also lacks robust, population-based estimates of safe household sharps disposal among insulin-treated adults, standardized metrics to differentiate pen needle from syringe reuse, and prospective evaluations linking targeted education and disposal access to patient and community-safety outcomes (4, 24). Furthermore, structural constraints—limited availability of community sharps-collection terminals and inconsistent reimbursement for pen needles—remain untested as modifiable levers in pragmatic policy trials. Therefore, this cross-sectional study of insulin-treated adults across China quantifies disposal pathways, prevalence of needle reuse, and community-safety outcomes while identifying multilevel determinants of unsafe practices through comprehensive risk factor analysis.

## 2 Methods

### 2.1 Study design and overview

This investigation employed a cross-sectional analytical design to characterize sharps disposal behaviors, needle-reuse practices, and community-safety outcomes among adults with insulin-treated diabetes in China. The study used a structured, interviewer-administered questionnaire with defined recall windows to quantify behaviors and exposures and to estimate associations with unsafe disposal, needle reuse, and community-safety incidents.

### 2.2 Setting and participants

This study was conducted in the Outpatient Department, The Central Hospital of Enshi Tujia and Miao Autonomous Prefecture (Enshi, Hubei, China). Data collection occurred between February 2023 and December 2024. Patients’ inclusion criteria were as follows: (1) Age ≥18 years; (2) clinician-confirmed diagnosis of diabetes mellitus (type 1, type 2, or other specified type); (3) self-administration of insulin via pen needles, syringes, or insulin pump within the past 30 days; (4) community-dwelling (non-institutionalized); (5) able to provide informed consent; and (6) sufficient cognitive capacity to complete the questionnaire as determined by the interviewer.

However, the exclusion criteria was: (1) Age <18 years; (2) no insulin use in the preceding 30 days; (3) insulin administered exclusively by healthcare professionals or caregivers (no self-injection); (4) institutionalized patients (e.g., nursing homes, long-term care facilities); (5) unable or unwilling to provide informed consent; (6) significant cognitive impairment precluding reliable recall; and (7) participation in other diabetes intervention studies that might influence disposal or injection behaviors.

### 2.3 Sampling strategy and sample size

Sampling frame and selection. A clinic-based consecutive sampling approach was used. All eligible outpatients presenting during staffed survey sessions were approached in sequence after clinical triage; recruitment rotated across weekdays and clinic sessions to minimize time-of-day selection bias. No cluster sampling was required because the study was a single-center study. A screening log recorded the numbers eligible, approached, consented, and declined to permit calculation of overall response and cooperation rates; reasons for non-participation were noted when offered.

**Figure 1:**
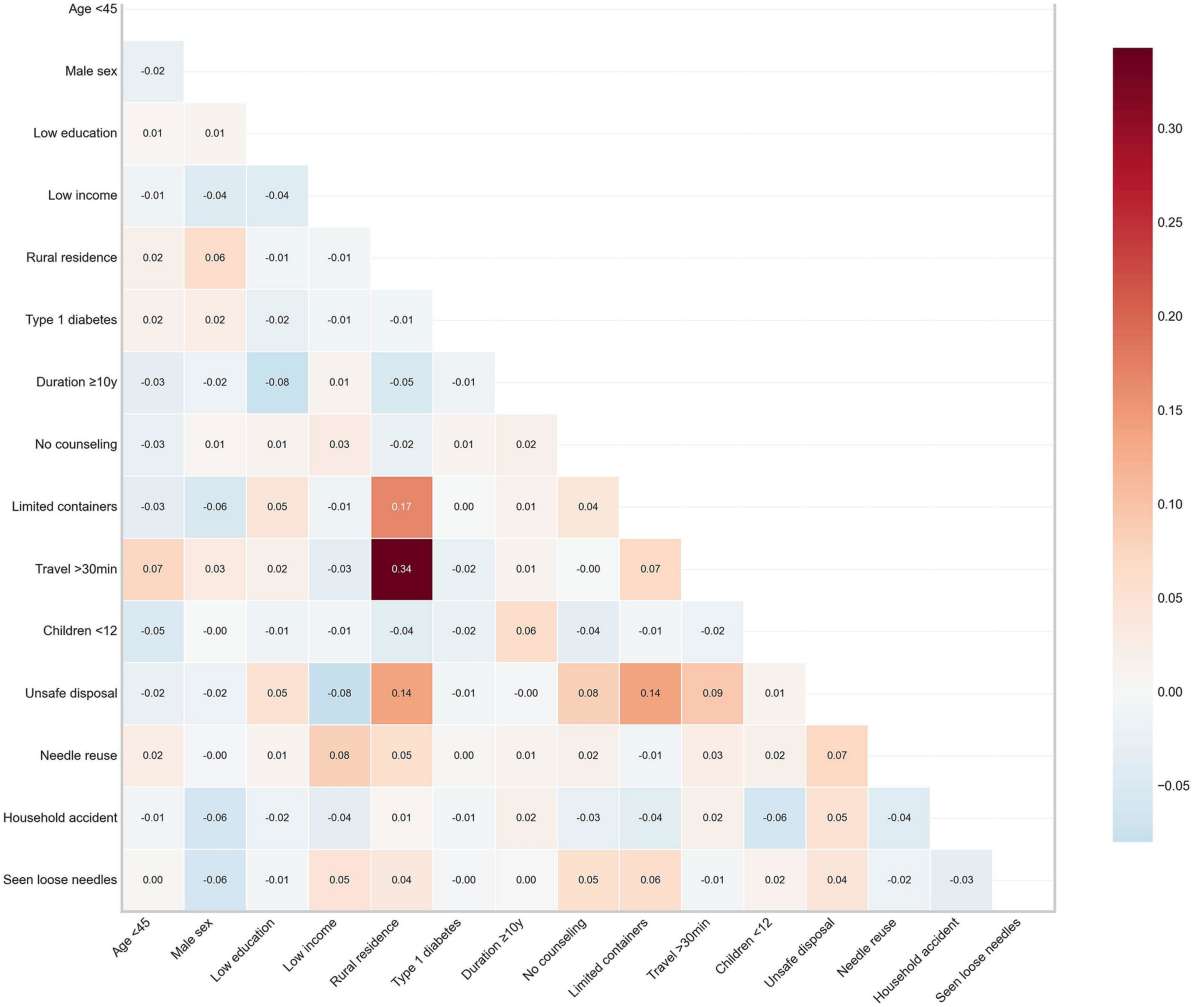
Risk factor correlation matrix. Heat map displaying Pearson correlation coefficients between key risk factors and safety outcomes. Correlation strength is indicated by color intensity (dark red = strongest positive correlation; dark blue = strongest negative correlation). Notable correlations include travel time >30 min with rural residence (*r* = 0.34) and limited container availability with rural residence (*r* = 0.17). Matrix demonstrates modest interdependencies among risk factors while confirming the appropriateness of the multivariable modeling approach, with the highest correlation coefficient (0.34) below collinearity concern thresholds.

Sample size adequacy. Sample size planning for adequate events-per-variable (EPV) in multivariable logistic models. With *N* = 1,810 and observed outcome prevalences (unsafe disposal 59.0%; needle reuse 32.5%), EPV far exceeded conventional thresholds (≥10) for the two primary models (unsafe disposal: 1,067 events ≈ > 60 EPV; needle reuse: 588 events ≈ > 30 EPV given ≈ 16 parameters). For community-safety endpoints, event counts were lower (household needlestick: 101; observed loose needles: 132); the corresponding EPV (~7 and ~9, respectively) informed parsimonious model specification and cautious interpretation. Variance inflation factors (VIF) were calculated for all predictor variables in multivariable logistic regression models using the vif() function from the car package. VIF values ranged from 1.08 to 2.87 across all predictors, well below the commonly accepted threshold of 10 (and below the conservative threshold of 5), indicating no substantial multicollinearity. The highest VIF values were observed for the correlation between lower education and low income (VIF = 2.87 for income, 2.54 for education), which remained within acceptable limits. A correlation matrix of all predictors showed the maximum pairwise correlation of *r* = 0.34 (travel time >30 min with rural residence), confirming model stability (see Figure 1).

### 2.4 Questionnaire development and structure

The instrument was developed from the empirical literature on injection technique and community sharps disposal and then adapted for local terminology and disposal pathways. Cross-cultural adaptation followed standard procedures: forward translation by a bilingual clinician, independent back-translation by a second translator blinded to the original, reconciliation by an expert panel (diabetes nurse educator, endocrinologist, public-health researcher), and cognitive interviewing with a small convenience sample of insulin-treated adults to ensure comprehension and relevance. Pilot field testing in the outpatient setting verified skip logic, recall wording, and response distributions; minor refinements in wording and ordering followed. Because the questionnaire comprised single-item behavioral measures rather than multi-item scales, internal consistency metrics (e.g., Cronbach’s alpha) were not applicable. Items were organized into sections capturing (i) demographics and socioeconomic position; (ii) clinical profile and insulin administration mode; (iii) sharps disposal behaviors and safety practices (30-day recall); (iv) needle-reuse behaviors, reasons, and supply availability (30-day recall); (v) knowledge, attitudes, and access to counseling and programs; and (vi) community-safety outcomes (12-month recall or 3-month recall per outcome). Response options and coding schemes were prespecified and are detailed below. “Prefer not to answer” and “not sure” options were provided where appropriate and treated as missing in the analysis.

### 2.5 Measures

#### 2.5.1 Primary outcomes

1. Unsafe sharps disposal (30-day recall). The primary disposal behavior was ascertained by asking, “In the past 30 days, how did you usually dispose of your used needles, lancets, or other sharps?” Response options included: (a) approved sharps container; (b) thick household container with secure sealing (e.g., detergent bottle); (c) pharmacy/clinic take-back program; (d) household trash (loose); (e) public bin; (f) toilet/sink; and (g) other (specify). For analysis, a composite binary indicator, unsafe disposal, was coded 1 for options (d)–(f) and coded 0 otherwise. A complementary safe disposal composite was coded 1 for options (a)–(c). The recall window and composite definitions align with public-health guidance on community sharps risk.
2. Any needle reuse (30-day recall). Participants reported the number of separate injections performed with the same needle during the past 30 days. A binary indicator of any reuse was coded 1 if the participant used any needle for ≥2 injections and coded 0 for single use only. Two descriptive intensities were computed: average injections per needle with the categories 1, 2, 3–4, and ≥5; and maximum reuse with the categories 1, 2, 3–4, 5–9, and ≥10.

#### 2.5.2 Secondary outcomes

Community- safety incidents: Three outcomes were assessed: (i) household needlestick injury among any household member in the past 12 months (yes/no); (ii) observed loose needles in community areas in the past 3 months (yes/no); and (iii) known waste-worker injury attributed to the participant’s sharps in the past 12 months (yes/no). Incidents were defined by self-report following a standard description of what constitutes a needlestick injury.

Safety practices: Participants reported whether a puncture-resistant container was used at the last disposal event (yes/no) and indicated the frequency of needle recapping prior to disposal on a 5-point scale (never, rarely, sometimes, often, and always).

### 2.6 Exposures and covariates

#### 2.6.1 Sociodemographic variables

Age was categorized as <30, 30–44, 45–59, and ≥60 years (reference in modeling). Sex was recorded as female, male, or intersex; modeling used female as the reference. Education was collected as no formal schooling, primary, secondary, higher secondary, college/university, and graduate or higher; for regression, the reference category was college/university+ (college/university or higher). Household income was reported as low, lower middle, upper middle, or high incomes; high income was used as the reference.

The type of residence (urban, peri-urban, and rural) and the presence of children <12 years in the household (yes/no) were recorded to contextualize disposal risk. Items on informal housing and pet access to waste were additionally documented for community-safety models where applicable.

#### 2.6.2 Clinical variables

Diabetes type (type 1, type 2, other) and diabetes duration in years were collected; a binary indicator duration ≥10 years was created for modeling. Insulin regimen (basal-only, basal–bolus, premixed, and pump) and injection device (pen with disposable needles, syringe and vial, pump infusion set) were recorded; a binary indicator insulin pump (vs. pen needles) was used in models of disposal and reuse.

#### 2.6.3 Access, knowledge, and program exposure

Participants reported the following: (i) receipt of counseling on sharps disposal from a healthcare professional in the past 12 months (yes/no); (ii) awareness of any pharmacy/clinic take-back program (yes/no); (iii) typical travel time from home to the nearest safe disposal location (≤10, 11–30, >30 min); and (iv) container availability at home or places of injection with options “readily available,” “sometimes available,” or “not available.” For regression, limited availability was coded 1 for “sometimes” or “not available,” and coded 0 for “readily available.”

#### 2.6.4 Knowledge and attitudes

Agreement with the statement “It is safe to dispose of used needles in regular trash if the needle is capped” was measured on a 5-point Likert scale (from “strongly agree” to “strongly disagree”) to describe normative beliefs regarding household disposal. Supply availability and reasons for reuse. Participants indicated the number of days in the past 30 days without sufficient sterile needles (0, 1–2, 3–7, 8–14, >14 days). For those reporting any reuse, a multiple-response item captured reasons for reuse, including cost concerns, convenience, stockouts/limited availability, forgetting to carry new needles, perceived safety (“still sharp/clean”), and environmental concerns.

### 2.7 Data collection procedures

Trained surveyors administered the instrument in Mandarin (with regionally appropriate terms for waste pathways) using a standardized script and visual show cards for disposal options and recall windows. Interviewers were trained to read questions verbatim, avoid leading prompts, and maintain a neutral stance; participants were seated away from accompanying family members, where possible, to reduce social-desirability influences. For sensitive items (e.g., disposal method, reuse), respondents could mark answers privately on a tablet and return it to the interviewer (sealed-screen mode). When self-administration was preferred, participants completed an identical electronic form with mandatory fields for key outcomes to minimize missing data. Logic checks prevented inconsistent responses (e.g., indicating no insulin use but reporting a disposal method). Recall aids (e.g., calendar prompts for “past 30 days” and examples of disposal locations) were used to reduce recall error.

### 2.8 Data management and coding

Data were captured electronically with range checks and stored in a secure database. Category labels were harmonized before analysis. For descriptive statistics, percentages were calculated from valid responses only, excluding “prefer not to answer” and “not sure.” Composite indicators (safe disposal, unsafe disposal) were generated as defined above. Multiple-response items (reasons for reuse) were represented as separate binary indicators. Continuous summary variables were not created beyond the prespecified categorical intensities for reuse.

### 2.9 Bias minimization

Several procedures mitigated common sources of bias. Social desirability: neutral scripts, optional private response entry, and separation from family members during sensitive questions were used to reduce reporting of unsafe behaviors. Recall bias: show cards, calendar anchors, and concrete examples were provided for 30-day and 12-month recall periods. Selection bias: a screening log documented eligibility, approach, consent, and completion to quantify response and cooperation rates; comparisons of basic demographics between participants and non-participants (where available) were planned to assess representativeness.

### 2.10 Statistical analysis

Descriptive statistics reported counts and percentages from valid responses, excluding “prefer not to answer”/“not sure”; multiple-response percentages were calculated among reuse reporters (*n* = 588) and travel-time percentages among respondents with known times (*n* = 1,732). Bivariate associations with unsafe disposal were tested using two-sided *χ*^2^ (*p* < 0.05). Multivariable logistic regression yielded adjusted odds ratios [aORs; 95% confidence interval (CIs)] for unsafe disposal and any needle reuse, adjusting for age, sex, education, income, diabetes type, diabetes duration ≥10 years, device (pump vs. pen), counseling, travel time (>30 vs. ≤30 min), take-back awareness, container availability, residence, and regimen; model performance: area under the receiver operating characteristic (ROC) curve (AUC) 0.78 and 0.74 with good calibration (Hosmer–Lemeshow test, *p* = 0.24, 0.41); interaction terms were not retained. Community-safety outcomes—household needlestick (12-month) and observed loose needles (3-month)—were modeled with exposures of unsafe disposal, any reuse, and recapping always/often, adjusted for demographics/clinical factors plus household (children <12, pets with waste access, vision/hand difficulties) and geography (rural residence, informal housing); AUCs 0.73 and 0.69 with adequate calibration (*p* > 0.05). Missing data were minimal; models used listwise deletion and were robust in sensitivity checks (redefining the safe-disposal composite, re-categorizing travel time, restricting to pen needle users); residence-type subgroups assessed heterogeneity. All analyses were conducted using R statistical software version 4.3.1 (R Foundation for Statistical Computing, Vienna, Austria). Specific packages included: stats (base statistical functions), car version 3.1–2 (variance inflation factor calculation), pROC version 1.18.4 (ROC curve analysis and AUC calculation), ResourceSelection version 0.3–6 (Hosmer–Lemeshow goodness-of-fit tests), ggplot2 version 3.4.3 (data visualization), and dplyr version 1.1.2 (data manipulation).

## 3 Results

### 3.1 Participant characteristics

Among 1,810 insulin-treated adults, the largest age stratum was 45–59 years (40.4%), followed by ≥60 years (32.3%), 30–44 years (22.0%), and <30 years (5.3%); 55.5% were male, 43.6% female, and 0.9% intersex. Educational attainment was heterogeneous: 23.8% reported college/university education and 5.9% graduate or higher education, whereas 15.0% had primary or no formal schooling. Household income skewed toward lower middle (38.4%) and low (28.2%) tiers. Most participants resided in urban areas (61.6%), and 35.8% reported children <12 years in the household. Clinically, type 2 diabetes predominated (85.3%); diabetes duration was evenly distributed across 5–9 years (26.4%), 10–14 years (26.5%), and ≥15 years (25.2%). Premixed insulin (41.8%) and basal-bolus (34.0%) regimens were common; most used pens with disposable needles (85.4%). These distributions are detailed in Table 1.

**Table 1:** Participant characteristics (*N* = 1,810).

Characteristic	Number (%)
Demographics	
Age, years	
<30	96 (5.3)
30–44	399 (22.0)
45–59	731 (40.4)
≥60	584 (32.3)
Sex	
Female	784 (43.6)
Male	997 (55.5)
Intersex	16 (0.9)
Education level	
No formal schooling	61 (3.4)
Primary	211 (11.9)
Secondary	593 (33.4)
Higher secondary	384 (21.6)
College/university	423 (23.8)
Graduate or higher	104 (5.9)
Household income level	
Low	501 (28.2)
Lower middle	683 (38.4)
Upper middle	475 (26.7)
High	119 (6.7)
Residence type	
Urban	1,115 (61.6)
Peri-urban	459 (25.4)
Rural	236 (13.0)
Children <12 years in household	638 (35.8)
Clinical characteristics	
Diabetes type	
Type 1	216 (12.1)
Type 2	1,522 (85.3)
Other	47 (2.6)
Diabetes duration	
<5 years	386 (21.9)
5–9 years	467 (26.4)
10–14 years	468 (26.5)
≥15 years	445 (25.2)
Insulin regimen	
Basal only	370 (20.7)
Basal-bolus	607 (34.0)
Premixed	747 (41.8)
Insulin pump	62 (3.5)
Injection device	
Pen with disposable needles	1,532 (85.4)
Syringe and vial	166 (9.3)
Pump infusion set	95 (5.3)

Data presented as a number (percentage). Percentages calculated from valid responses excluding “prefer not to answer” and “not sure” responses.

### 3.2 Sharps disposal practices and safety behaviors

**Figure 2:**
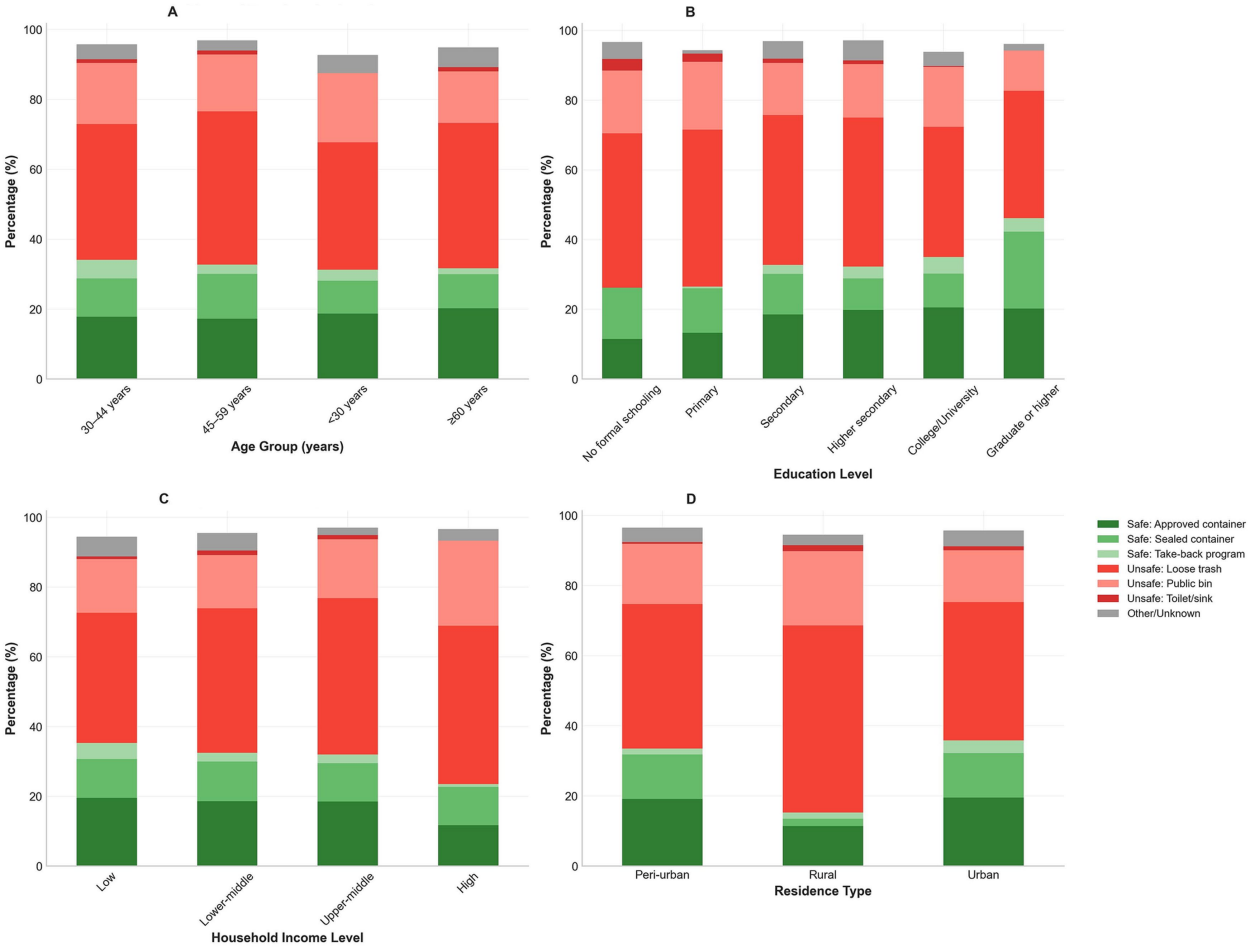
Sharps disposal practices across demographic groups. Stacked bar charts showing distribution of sharps disposal methods stratified by demographic characteristics. **(A)** Disposal practices by age group demonstrate higher unsafe disposal rates among younger participants. **(B)** Disposal practices by education level, showing educational gradients in safety behaviors. **(C)** Disposal practices by income level, illustrating socioeconomic disparities. **(D)** Disposal practices by residence type, highlighting rural–urban differences.

Household trash disposal (loose) was the most frequent method (43.5%; 95% CI: 41.2–45.8%), followed by public bins (15.9%; 95% CI: 14.3–17.7%), whereas approved sharps containers were used by 24.7% (95% CI: 22.8–26.7%), sealed thick household containers and pharmacy/clinic take-back programs accounted for 5.5 and 3.0%, respectively. Only 35.0% (95% CI: 32.8–37.2%) reported using a puncture-resistant container at the last disposal event, and recapping was common (always/often: 32.9%; 95% CI: 30.8–35.1%). The composite indicators showed 59.0% (95% CI: 56.7–61.2%) engaged in unsafe disposal and 32.6% (95% CI: 30.5–34.8%) in safe disposal. These patterns are summarized in Table 2 and are consistent with the demographic-stratified stacks (as shown in Figures 2A–D and Table 2).

**Table 2:** Sharps disposal practices and safety behaviors (*N* = 1,810).

Practice	Number (%)
Usual disposal method (past 30 days)	
Approved sharps container	440 (24.7)
Thick household container (sealed)	97 (5.5)
Household trash (loose)	775 (43.5)
Pharmacy/clinic take-back	53 (3.0)
Public bin	284 (15.9)
Toilet/sink	8 (0.4)
Other	128 (7.2)
Safety practices (past 30 days)	
Used a puncture-resistant container (last disposal)	623 (35.0)
Recapping needles before disposal	
Never	448 (24.8)
Rarely	192 (10.6)
Sometimes	573 (31.7)
Often	361 (19.9)
Always	236 (13.0)
Composite safety indicators	
Safe disposal practices^a^	590 (32.6)
Unsafe disposal practices^b^	1,067 (59.0)

a Safe disposal includes an approved sharps container, a thick household container (sealed), or a pharmacy/clinic take-back program.

b Unsafe disposal includes household trash (loose), public bins, or toilets/sink disposal.

### 3.3 Needle reuse behaviors and supply availability

**Figure 3:**
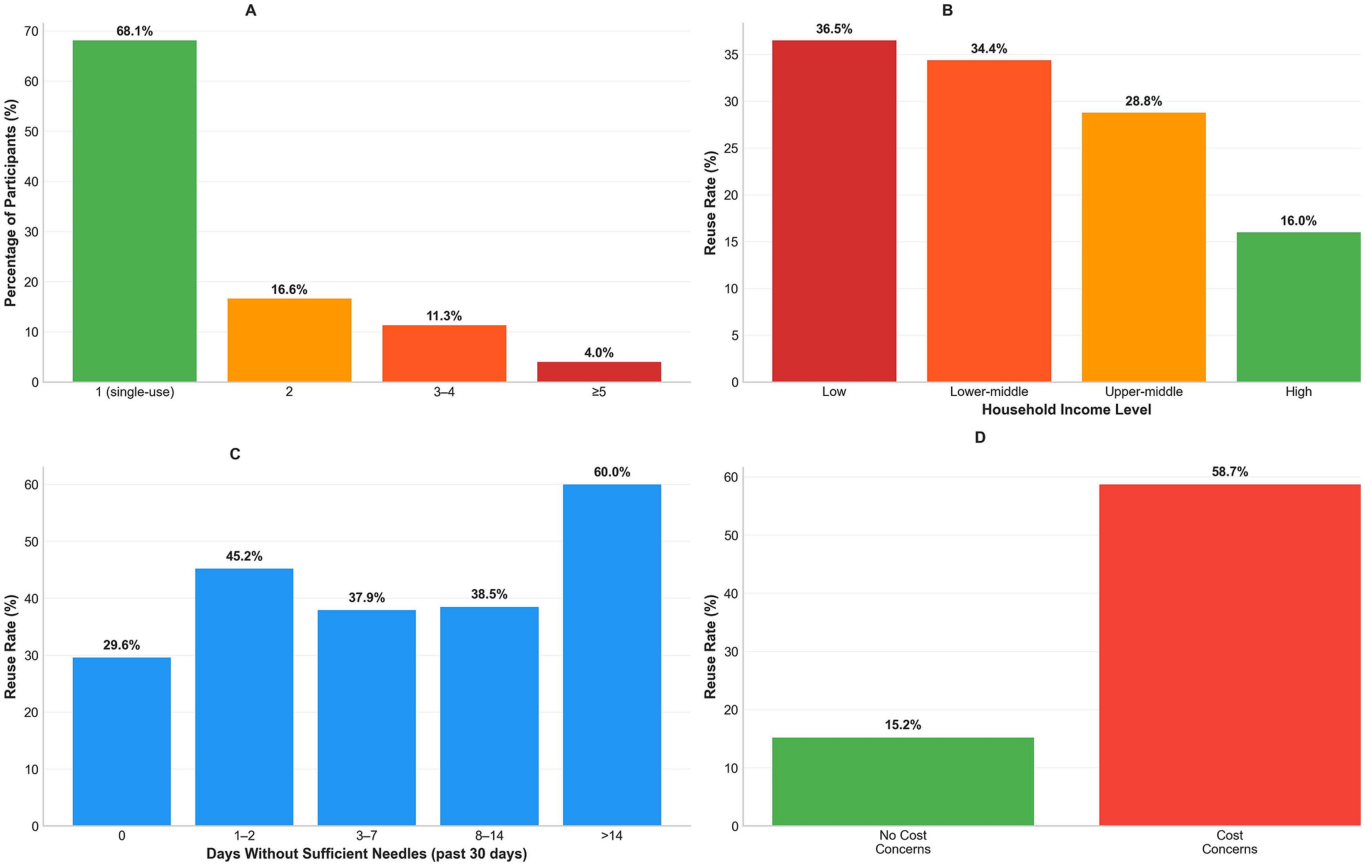
Needle reuse patterns and associated risk factors. **(A)** Average injections per needle distribution (68.1% single-use needles, 4.0% ≥ 5 uses). **(B)** Reuse rates by income (36.5% low to 16.0% high income). **(C)** Reuse rates by supply shortage duration (29.6% no shortage to 60.0% > 14 days). **(D)** Reasons for reuse among 588 users (cost 47.1%, convenience 35.5%, supply issues 31.3%).

Any needle reuse in the preceding 30 days was reported by 32.5% of participants (95% CI: 30.4–34.7%). Average injections per needle clustered at single use (68.1%), with additional strata at 2 (16.6%), 3–4 (11.3%), and ≥5 (4.0%); maximum reuse reached ≥10 injections in 2.8%. Among reusers (n = 588), cost concerns (47.1%), convenience (35.5%), and stockouts/limited availability (31.3%) were the leading reasons; 20.4% forgot to carry new needles, and 18.0% perceived reused needles as “still sharp/clean.” Supply constraints were non-trivial: 45.7% reported ≥1 day in the prior 30 days without sufficient sterile needles, including 7.9% reporting ≥8 days. These data are presented in Table 3 and depicted as reuse intensity and reason distributions (as shown in Figures 3A–D and Table 3). Differences between “no needle reuse” (67.5%) and “single use” (68.1%) reflect distinct valid-response denominators for the corresponding items.

**Table 3:** Needle reuse behaviors and associated factors (*N* = 1,810).

Behavior/factor	Number (%)
Needle reuse practices (past 30 days)	
Any needle reuse	588 (32.5)
No needle reuse	1,220 (67.5)
Average injections per needle (past 30 days)	
1 (single-use needles)	1,220 (68.1)
2	297 (16.6)
3–4	203 (11.3)
≥5	71 (4.0)
Maximum needle reuse (past 30 days)	
1 (single-use-only needles)	1,220 (68.6)
2	235 (13.2)
3–4	179 (10.1)
5–9	95 (5.3)
≥10	49 (2.8)
Reasons for needle reuse^a^ (*n* = 588)	
Cost concerns	277 (47.1)
Convenience	209 (35.5)
Stockouts or limited availability	184 (31.3)
Forgot to carry new needles	120 (20.4)
Perceived safety (“still sharp/clean”)	106 (18.0)
Environmental concerns	75 (12.8)
Supply availability	
Days without sufficient sterile needles (past 30 days)	
0	967 (54.3)
1–2	419 (23.5)
3–7	254 (14.3)
8–14	95 (5.3)
>14	47 (2.6)

a Multiple responses possible. Percentages calculated among participants who reported any needle reuse (*n* = 588).

### 3.4 Community-safety outcomes, knowledge, and access

**Figure 4:**
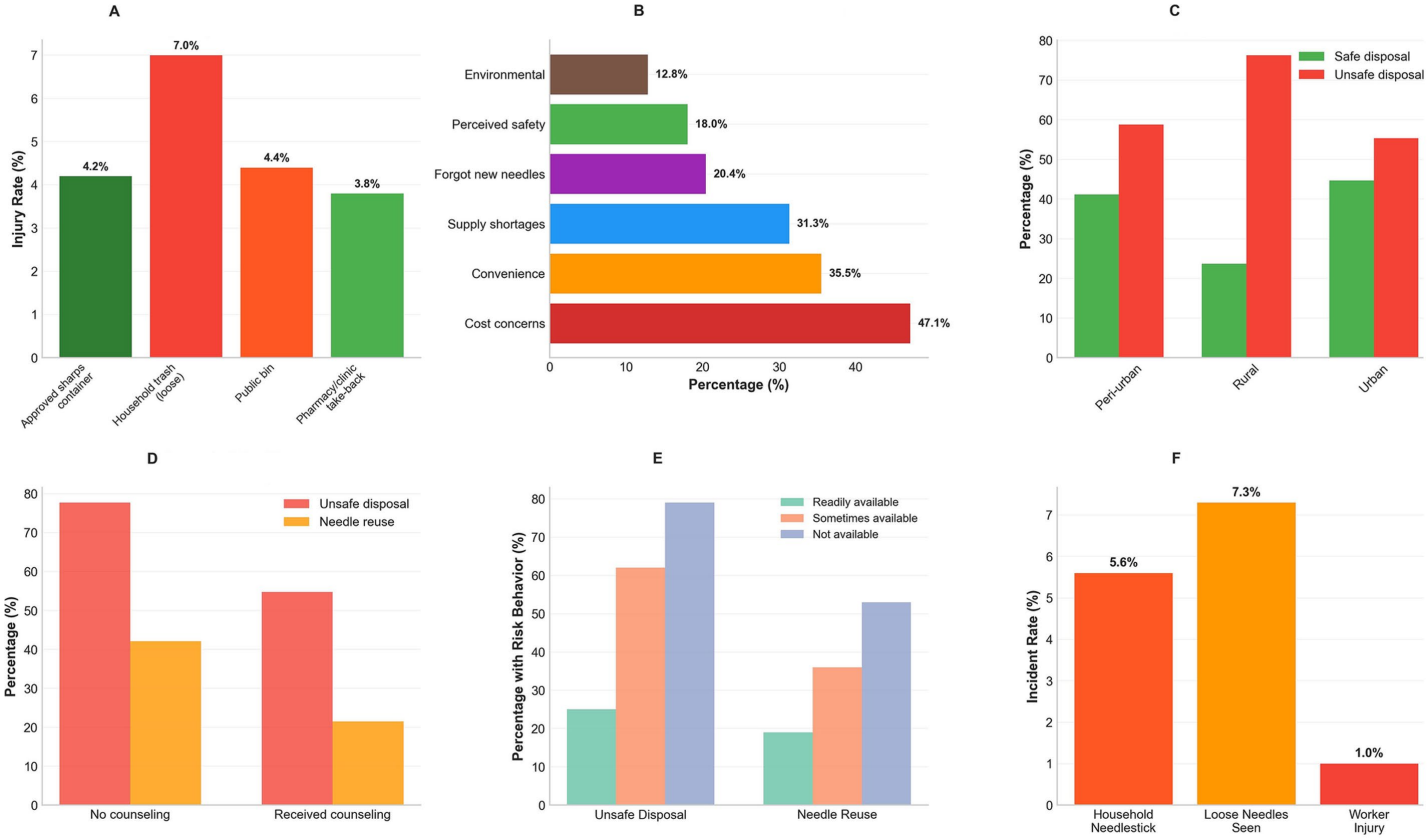
Community-safety outcomes and risk behavior patterns. **(A)** Household needlestick rates by disposal method (7.0% loose trash vs. 4.2% approved containers). **(B)** Reuse reasons ranked by frequency. **(C)** Safe disposal by geographic location. **(D)** Healthcare counseling impact on risk behaviors. **(E)** Container availability affects practices. **(F)** Community incident rates (5.6% household, 7.3% loose needles, 1.0% worker injury).

Household needlestick injury over 12 months occurred in 5.6% of households (95% CI: 4.6–6.8%), 7.3% observed loose needles in community areas over 3 months (95% CI: 6.2–8.6%), and 1.0% reported a known waste worker injury attributable to their sharps (95% CI: 0.6–1.6%). Nearly half agreed that it is safe to discard capped needles in regular trash (strongly agree/agree 49.8%), whereas 35.1% disagreed/strongly disagreed; 45.0% had received sharps-disposal counseling in the prior 12 months, 50.4% were aware of take-back programs, and travel time to the nearest safe-disposal location exceeded 30 min for 21.7%. These findings are summarized in Table 4; incident rate panels are shown in the community-safety figure (as shown in Figures 4A,F and Table 4).

**Table 4:** Community-safety outcomes and knowledge (*N* = 1,810).

Outcome/knowledge area	Number (%)
Safety incidents	
Accidental needlestick injury in the household (past 12 months)	101 (5.6)
Observed loose needles in community areas (past 3 months)	132 (7.3)
Known waste worker injury from patient’s sharps (past 12 months)	19 (1.0)
Knowledge and attitudes	
Agreement: “Safe to dispose in regular trash if capped”	
Strongly agree	361 (19.9)
Agree	542 (29.9)
Neither agree nor disagree	271 (15.0)
Disagree	361 (19.9)
Strongly disagree	275 (15.2)
Healthcare and program access	
Received sharps disposal counseling from a healthcare professional (past 12 months)	814 (45.0)
Aware of the pharmacy/clinic take-back program	904 (50.4)
Travel time to the safe disposal location	
≤10 min	452 (26.1)
11–30 min	904 (52.2)
>30 min	376 (21.7)

Data presented as a number (percentage). Travel time percentages calculated from participants with known travel times (*n* = 1,732).

### 3.5 Bivariate associations with unsafe disposal

Unsafe disposal varied significantly by age, sex, education, income, diabetes type, and counseling. Younger groups exhibited higher unsafe disposal (e.g., <30 years: 77.1% vs. ≥60 years: 65.9%; *χ*^2^ = 18.7; *p* < 0.001). Men had a higher rate of unsafe disposal than women (70.2% vs. 63.3%; *χ*^2^ = 12.4; *p* < 0.001). A strong educational gradient was observed (no formal/primary education: 80.1% vs. college/university and above education: 57.5%; *χ*^2^ = 45.2; *p* < 0.001), alongside an income gradient (low: 76.1% vs. high: 47.9%; *χ*^2^ = 38.9; *p* < 0.001). Type 2 diabetes was associated with more unsafe disposal than type 1 (67.9% vs. 60.2%; *χ*^2^ = 8.3; *p* = 0.016). Counseling was protective (unsafe disposal 54.8% with counseling vs. 77.7% without; *χ*^2^ = 89.4; *p* < 0.001). These associations are shown in Table 5 and displayed in demographic stacks (as shown in Figures 2A–D and Table 5).

**Table 5:** Bivariate associations with unsafe disposal practices.

Characteristic	Safe disposal	Unsafe disposal	χ^2^ statistic	*p*-value
	Number (%)	Number (%)		
Demographics				
Age group, years			18.7	<0.001
<30	22 (22.9)	74 (77.1)		
30–44	108 (27.1)	291 (72.9)		
45–59	261 (35.7)	470 (64.3)		
≥60	199 (34.1)	385 (65.9)		
Sex			12.4	<0.001
Female	288 (36.7)	496 (63.3)		
Male	297 (29.8)	700 (70.2)		
Education level			45.2	<0.001
No formal/Primary	54 (19.9)	218 (80.1)		
Secondary	178 (30.0)	415 (70.0)		
Higher secondary	134 (34.9)	250 (65.1)		
College/university+	224 (42.5)	303 (57.5)		
Household income			38.9	<0.001
Low	120 (23.9)	381 (76.1)		
Lower middle	198 (29.0)	485 (71.0)		
Upper middle	190 (40.0)	285 (60.0)		
High	62 (52.1)	57 (47.9)		
Clinical factors				
Diabetes type			8.3	0.016
Type 1	86 (39.8)	130 (60.2)		
Type 2	488 (32.1)	1,034 (67.9)		
Healthcare counseling (past 12 months)			89.4	<0.001
Yes	368 (45.2)	446 (54.8)		
No	222 (22.3)	774 (77.7)		

Safe disposal is defined as an approved sharps container, sealed thick container, or pharmacy take-back. Chi-square tests were performed for categorical associations. *p* values <0.05 are considered statistically significant.

### 3.6 Multivariable models for primary outcomes

**Figure 5:**
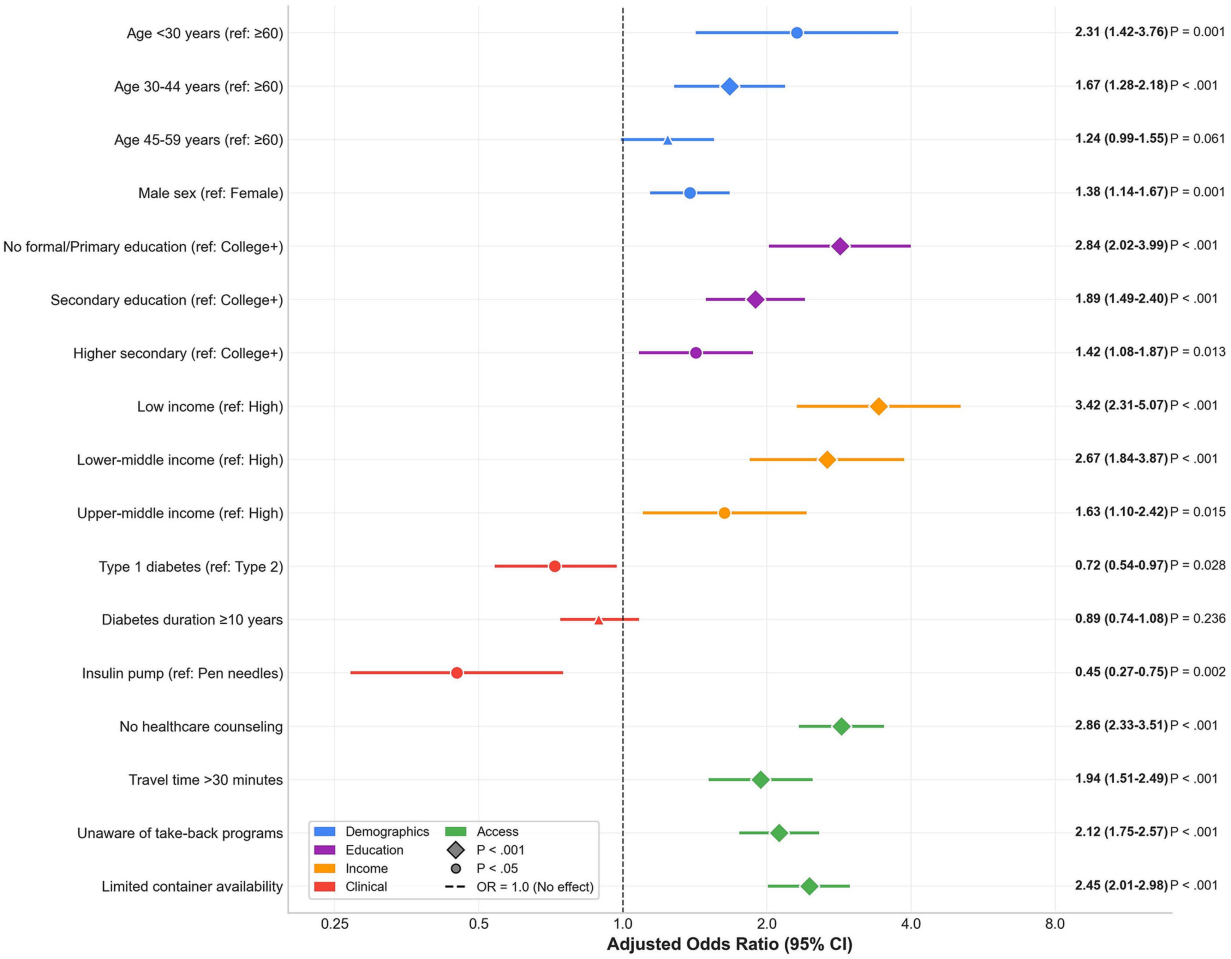
Multivariable logistic regression: risk factors for unsafe disposal practices. Forest plot displaying adjusted odds ratios (aORs) and 95% confidence intervals for factors associated with unsafe sharps disposal practices among 1,810 insulin-treated adults with diabetes. Reference categories are shown in parentheses. Color coding indicates variable domains: demographics (blue), education (purple), income (orange), clinical factors (red), and access factors (green). Model performance: AUC = 0.78, Hosmer–Lemeshow test, *p* = 0.24.

In the adjusted model for unsafe disposal, higher odds were observed for younger age (<30 years, aOR: 2.31; 95% CI: 1.42–3.76; 30–44 years, aOR: 1.67; 95% CI: 1.28–2.18), male sex (aOR: 1.38; 1.14–1.67), lower education (no formal/primary education: aOR: 2.84; 95% CI: 2.02–3.99; secondary education: aOR: 1.89; 95% CI: 1.49–2.40; higher secondary education: aOR: 1.42; 95% CI: 1.08–1.87), lower income (low income: aOR: 3.42; 95% CI: 2.31–5.07; lower middle income: aOR: 2.67; 95% CI: 1.84–3.87; upper middle income: aOR: 1.63; 95% CI: 1.10–2.42), longer travel time (>30 min: aOR: 1.94; 95% CI: 1.51–2.49), lack of awareness regarding take-back programs (aOR: 2.12; 95% CI: 1.75–2.57), and limited container availability (aOR: 2.45; 95% CI: 2.01–2.98). Lower odds were observed for type 1 diabetes (aOR: 0.72; 95% CI: 0.54–0.97), insulin pump use (aOR: 0.45; 95% CI: 0.27–0.75), and receipt of counseling (aOR: 0.35; 95% CI: 0.29–0.43). Model performance was strong (AUC: 0.78; Hosmer–Lemeshow test, *p* = 0.24). In the adjusted model for any needle reuse, higher odds were observed for younger age (<30 years: aOR: 1.89; 95% CI: 1.18–3.03; 30–44 years: aOR: 1.52; 95% CI: 1.19–1.94), male sex (aOR: 1.29; 95% CI: 1.07–1.55), lower education (no formal/primary education: aOR: 2.16; 95% CI: 1.56–2.99; secondary education: aOR: 1.67; 95% CI: 1.33–2.10; higher secondary education: aOR: 1.31; 95% CI: 1.01–1.70), lower income (low income: aOR: 2.87; 95% CI: 1.95–4.24; lower middle income: aOR: 2.24; 95% CI: 1.56–3.22; upper middle income: aOR: 1.45; 95% CI: 0.99–2.12), longer duration (≥10 years: aOR: 1.34; 95% CI: 1.11–1.61), longer travel time (aOR: 1.78; 95% CI: 1.39–2.28), lack of awareness regarding take-back programs (aOR: 1.87; 95% CI: 1.55–2.26), and limited container availability (aOR: 3.21; 95% CI: 2.64–3.90). Lower odds were observed with insulin pump use (aOR: 0.31; 95% CI: 0.15–0.64) and receipt of counseling (aOR: 0.42; 95% CI: 0.35–0.51). Model performance was adequate (AUC: 0.74; Hosmer–Lemeshow test, *p* = 0.41). These adjusted estimates are presented in Table 6; the unsafe-disposal model is visualized as a forest plot (as shown in Figure 5 and Table 6).

**Table 6:** Multivariable logistic regression models for primary outcomes.

Variable	Unsafe disposal	*p*-value	Needle reuse	*p*-value
	Adjusted OR (95% CI)		Adjusted OR (95% CI)	
Demographics				
Age group (reference: ≥60 years)				
<30 years	2.31 (1.42–3.76)	0.001	1.89 (1.18–3.03)	0.008
30–44 years	1.67 (1.28-–2.18)	<0.001	1.52 (1.19–1.94)	0.001
45–59 years	1.24 (0.99–1.55)	0.061	1.18 (0.95–1.46)	0.134
Male sex (reference: Female)	1.38 (1.14–1.67)	0.001	1.29 (1.07–1.55)	0.007
Education (reference: College/university+)				
No formal/primary	2.84 (2.02–3.99)	<0.001	2.16 (1.56–2.99)	<0.001
Secondary	1.89 (1.49–2.40)	<0.001	1.67 (1.33–2.10)	<0.001
Higher secondary	1.42 (1.08–1.87)	0.013	1.31 (1.01–1.70)	0.044
Income (reference: High)				
Low	3.42 (2.31–5.07)	<0.001	2.87 (1.95–4.24)	<0.001
Lower middle	2.67 (1.84–3.87)	<0.001	2.24 (1.56–3.22)	<0.001
Upper middle	1.63 (1.10–2.42)	0.015	1.45 (0.99-–2.12)	0.055
Clinical factors				
Type 1 diabetes (reference: Type 2)	0.72 (0.54–0.97)	0.028	0.83 (0.63–1.09)	0.182
Diabetes duration ≥10 years	0.89 (0.74–1.08)	0.236	1.34 (1.11–1.61)	0.002
Insulin pump use (reference: pen needles)	0.45 (0.27–0.75)	0.002	0.31 (0.15–0.64)	0.002
Access and knowledge				
Healthcare counseling received	0.35 (0.29–0.43)	<0.001	0.42 (0.35–0.51)	<0.001
Travel time >30 min	1.94 (1.51–2.49)	<0.001	1.78 (1.39–2.28)	<0.001
Unaware of take-back programs	2.12 (1.75–2.57)	<0.001	1.87 (1.55–2.26)	<0.001
Container availability limited	2.45 (2.01–2.98)	<0.001	3.21 (2.64–3.90)	<0.001

OR indicates odds ratio; CI, confidence interval. Adjusted for all variables shown and residence type and diabetes regimen. Model C-statistic (AUC): 0.78 for unsafe disposal, 0.74 for needle reuse. Hosmer–Lemeshow test: *p* = 0.24 (unsafe disposal), *p* = 0.41 (needle reuse).

### 3.7 Community-safety risk models

After adjustment, unsafe disposal was strongly associated with household needlestick injury (aOR 3.47; 95% CI: 2.01–5.99) and observing loose needles in community areas (aOR: 2.89; 95% CI: 1.85–4.52). Needle reuse also increased risks (household needlestick: aOR 2.23; 95% CI: 1.42–3.50); observed loose needles (aOR: 1.67; 95% CI: 1.13–2.47). Frequent recapping was associated with household needlestick (aOR: 1.89; 95% CI: 1.19–3.00) but not clearly with observed loose needles (aOR: 1.45; 0.98–2.15; *p* = 0.064). Household and geographic factors showed expected patterns: children <12 years (aOR: 2.67; 95% CI: 1.68–4.24) and informal housing (aOR: 2.45; 95% CI: 1.54–3.90) were associated with household needlestick; rural residence was not (aOR 1.23, 0.71–2.13). For observed loose needles, rural residence (aOR: 2.18; 95% CI: 1.42–3.35), informal housing (aOR: 3.67; 95% CI: 2.41–5.59), and pets with waste access (aOR: 1.56; 95% CI: 1.02–2.38) were associated with higher odds. Model discrimination was acceptable (AUC: 0.73 and 0.69) with adequate calibration (Hosmer––Lemeshow test, *p* > 0.05). These findings are summarized in Table 7, with descriptive counterparts in the community-safety panels (as shown in Figures 4A–F and Table 7).

**Table 7:** Risk factors for community-safety incidents.

Risk factor	Household needlestick	*p*-value	Observed loose needles	*p*-value
	Adjusted OR (95% CI)		Adjusted OR (95% CI)	
Disposal practices				
Unsafe disposal practices	3.47 (2.01–5.99)	<0.001	2.89 (1.85–4.52)	<0.001
Needle reuse	2.23 (1.42–3.50)	<0.001	1.67 (1.13–2.47)	0.01
Recapping always/often	1.89 (1.19–3.00)	0.007	1.45 (0.98–2.15)	0.064
Household factors				
Children <12 years present	2.67 (1.68–4.24)	<0.001	1.34 (0.90–1.99)	0.149
Pets with waste access	1.78 (1.09–2.91)	0.021	1.56 (1.02–2.38)	0.041
Vision/hand difficulties	1.94 (1.15–3.28)	0.013	1.23 (0.78–1.94)	0.372
Geographic factors				
Rural residence (reference: Urban)	1.23 (0.71–2.13)	0.46	2.18 (1.42–3.35)	<0.001
Informal housing	2.45 (1.54–3.90)	<0.001	3.67 (2.41–5.59)	<0.001

Models adjusted for age, sex, education, income, diabetes type, and duration. Household needlestick model C–statistic: 0.73. Community exposure model C-statistic: 0.69. Both models showed adequate calibration (Hosmer–Lemeshow test, *p* > 0.05).

## 4 Discussion

Current clinic-based cross-sectional study from Enshi, Hubei, shows that unsafe household sharps disposal is common (59.0%) and needle reuse persists (32.5%), with both behaviors independently linked to household needlestick injury and observed community needles (3, 25). These patterns accord with China-specific reports of limited counseling and disposal infrastructure (2, 4, 26); harm-reduction should prioritize puncture-resistant, leak-proof, rigid containers—for example, repurposed thick-walled detergent or juice bottles with secure caps—while avoiding cardboard, and expand convenient take-back options. Income constraints plausibly underline reuse: most respondents reported lower middle (38.4%) or low (28.2%) income, and only 6.7% reported high income, mirroring strong socioeconomic gradients. Disparities by sex and age were evident, with higher risk among men and younger adults, indicating the need for targeted messaging coupled with container provision and accessible return pathways. Collectively, these findings place the cohort at the upper end of global estimates and highlight actionable levers to improve patient and community safety (2, 18, 26).

These findings are broadly consistent with prior reports that have documented suboptimal community disposal practices among insulin users worldwide; yet, the magnitude observed here is higher than many estimates from health-system–embedded cohorts in high-income settings. For example, Montoya and colleagues, surveying US outpatients and inpatients, reported unsafe disposal of insulin needles by approximately one-third of participants, with sharp-to-trash discarding representing the dominant pathway (2). In contrast, a recent single-center study from China observed a markedly lower prevalence of safe disposal (10.3%), reflecting limited counseling and infrastructure as key constraints (26). A 2023 systematic review similarly concluded that household-generated sharps are frequently discarded in general waste streams across diverse settings, with education and awareness gaps repeatedly implicated (4, 18). In aggregate, these data situate the present study at the upper end of the global range for unsafe disposal, while aligning with China-specific evidence indicating structural and knowledge barriers.

The age, sex, education, and income gradients observed in the adjusted models are consonant with extant literature on health literacy, access, and risk tolerance. Lower educational attainment and lower income have repeatedly been associated with unsafe sharps handling, both in community cohorts and in clinic-based samples (2, 26, 27). Notably, lack of awareness regarding take-back programs and longer travel time to safe-disposal points were independently associated with unsafe disposal and with needle reuse in the current study, extending observations from U.S. and European initiatives that have linked density of collection sites and mail-back options with safer behaviors (2, 11, 28). The strength of these associations suggests that increasing the availability and salience of convenient, low-friction disposal pathways—including pharmacy drop-off, kiosk terminals, and mail-back—may yield measurable reductions in unsafe discarding.

The high prevalence of pen use in China provides an important context for reuse behaviors. National surveys and cross-country comparisons highlight near-universal insulin-pen adoption, widespread use of short (4–5 mm) pen needles, and persistent knowledge gaps regarding site rotation and needle reuse (18). Forum for Injection Technique & Therapy Expert Recommendations (FITTER)-aligned recommendations explicitly discourage reuse on the grounds of tip deformation, polymer fragmentation, and heightened risk of lipohypertrophy (LH), all of which can degrade dose reproducibility and absorption kinetics (5). In concordance with these mechanistic principles, Chinese randomized data demonstrate that structured injection-technique education—including avoidance of LH sites, consistent rotation, single-use needles, and correct insertion angles—reduces total daily insulin dose and improves glycemic stability (29, 30). The present study’s independent association between limited container availability and needle reuse plausibly reflects both cost-containment strategies and perceived inconvenience; that linkage has been indirectly supported by observational data indicating that reimbursement for pen needles is associated with lower overall costs and potentially better adherence to recommended technique (20, 21).

Moreover, the community-safety signals detected here are biologically and behaviorally plausible. Needlesticks in households cluster where recapping is frequent and where children or pets have access to waste streams, a pattern echoed by qualitative and surveillance studies in home healthcare environments (31–33). Public-space exposures have been described in pediatric and environmental health literatures, where discarded sharps pose low but non-zero risks of blood-borne pathogen transmission and substantial psychological distress, necessitating prophylaxis and follow-up (25, 34, 35). The present models identify unsafe disposal and reuse as independent predictors of both household injury and observed community sharps, reinforcing the concept that patient-level practices propagate community-level hazards.

At the same time, several contrasts with prior work merit discussion. First, while FITTER and subsequent reviews emphasize the contribution of LH to glucose variability and hypoglycemia (5, 36, 37), some recent analyses have questioned the short-term glycemic impact of limited reuse under controlled conditions, even as they acknowledge increased LH and tip deformity (38, 39). The current study did not quantify glycemic outcomes, but included clinical proxies (e.g., insulin-dose fidelity) and risk behaviors relevant to LH pathogenesis; thus, the interpretation centers on safety and community externalities rather than glycemic equivalence. Second, US cohorts have reported higher awareness of disposal options and greater use of approved containers or take-back channels than seen here (2); reflecting the policy infrastructure, including consolidated state guidance and pharmacy-based disposal solutions that reduce travel burden. Third, the single-center Chinese study referenced above found that instruction on sharps disposal and awareness of blood-borne pathogen risks were the predominant correlates of safe disposal (2); the present analysis extends those findings by quantifying additional access variables (travel time, container availability) and demonstrating independent associations with both unsafe disposal and reuse in multivariable models that adjust for socioeconomic position and clinical covariates.

The determinants identified here map closely to modifiable levers. Education and counseling, delivered by trained nurses, pharmacists, and diabetes educators, have repeatedly improved disposal knowledge and practices in community settings (4, 26). However, the current study shows that education alone is unlikely to be sufficient when physical access and costs are constraining; the strong effects of travel time and container scarcity argue for combined approaches that pair FITTER-concordant counseling with the provision of puncture-resistant containers and a clearly defined, convenient drop-off pathway. International experience with kiosk-based collection, pharmacy partnerships, and mail-back services indicates feasibility and acceptability, with attendant reductions in sharps found in municipal waste streams (2, 40, 41). In China, policy levers may include reimbursement for pen needles—as suggested by health services analyses linking coverage to lower high-cost events—and standardized municipal guidance on community sharps collection (8, 12–14).

Sex differences warrant further nuance. In adjusted analyses, male participants had higher odds of unsafe disposal (aOR: 1.38; 95% CI: 1.14–1.67) and needle reuse (aOR: 1.29; 95% CI: 1.07–1.55) than female participants. Potential mechanisms include greater frequency of out-of-home injections among men, lower perceived risk, and differences in caregiving roles and waste handling within households. Programmatically, tailoring messages for men—emphasizing the legal, environmental, and familial consequences of improper disposal, alongside easy-access container provision and clearly signposted drop-off points—may reduce these disparities. Similarly, younger adults showed higher adjusted odds of unsafe disposal and reuse, in line with research that associates younger age with lower perceived risk and higher mobility, which may increase out-of-home injections and opportunities for improper discarding (2, 4). In addition, male sex was also associated with greater odds of both outcomes; while mechanisms remain speculative, studies in injury epidemiology and risk-taking behaviors provide plausible explanatory frameworks. Targeted messaging for younger adults and men—emphasizing the legal, environmental, and familial implications of improper disposal—may therefore be warranted.

The association between insulin pump therapy and lower odds of unsafe disposal and reuse aligns with device-specific practice patterns. Pump users generate fewer detachable sharps events per week and have structured change protocols, which may reduce opportunities for *ad hoc* discard and reuse. This observation parallels prior reports that infusion-set education and scheduled supply replacement reduce risk behaviors and may indirectly protect household members and workers (4, 8). Conversely, longer diabetes duration was associated with needle reuse but not with unsafe disposal after adjustment, a pattern reported variably across studies and possibly reflecting habituation, cost constraints, or complacency regarding reuse hazards (4, 26, 42, 43).

Potential underestimation and policy cost-effectiveness implications. The observed needle reuse prevalence of 32.5% likely represents a conservative estimate due to social-desirability bias inherent in self-reported behaviors, particularly for practices known to deviate from clinical recommendations. If the actual reuse rate is substantially higher—potentially approaching 40–50% as suggested by indirect evidence from lipohypertrophy surveys in similar Chinese cohorts (4, 18)—the economic and clinical case for intervention becomes even more compelling. Specifically, underestimation would imply: (1) a larger target population for needle supply and education programs, thereby improving economies of scale and reducing per-capita intervention costs; (2) greater aggregate burden of preventable adverse outcomes (lipohypertrophy, dose variability, injection-site complications) that successful programs would avert; and (3) amplified community-safety externalities, as higher reuse rates correlate with increased needlestick risk exposure for household members and waste handlers. Economic modeling studies across diverse health systems indicate that provision of single-use needles, coupled with disposal infrastructure, can be cost-saving when accounting for avoided complications, emergency department visits, and occupational injuries (20, 21). If our prevalence estimates are indeed conservative, the return on investment for comprehensive sharps safety programs—encompassing reimbursement for pen needles, free container distribution, and accessible take-back systems—would exceed current projections. Future studies should employ triangulation methods (e.g., comparing self-reports with needle consumption records and clinical examination for lipohypertrophy) to bound true prevalence and inform more precise cost-effectiveness analyses for policy planning.

The cross-sectional design precludes definitive causal inference, yet several associations align with established causal pathways. The protective association between healthcare counseling and unsafe disposal (aOR: 0.35) and needle reuse (aOR: 0.42) accords with randomized evidence that structured education causally improves injection behaviors (29, 30). The association between limited container availability and unsafe practices (aOR: 2.45 for disposal, 3.21 for reuse) likely reflects both direct causal pathways and shared determinants (e.g., rural residence), as supported by experimental container provision programs (11, 28). Socioeconomic gradients may operate through cost barriers, health literacy, healthcare access, and infrastructure disparities—requiring longitudinal studies to decompose these pathways. The association between unsafe disposal and community incidents (household needlestick: aOR: 3.47; observed needles: aOR: 2.89) is biologically plausible but cannot exclude reverse causality. We interpret findings as identifying potentially modifiable risk factors; pragmatic trials embedding disposal education and infrastructure improvements into routine care are needed to establish causal effectiveness and cost-effectiveness.

Policy implications are immediate. Scaling community sharp-collection terminals, enabling pharmacy-based take-back, and integrating mail-back systems would directly address travel-time barriers (44, 45). Embedding disposal counseling and supply access into routine diabetes education, discharge planning, and pharmacy encounters would increase exposure to correct practices. Importantly, reimbursement policies for pen needles and containers could reduce economic incentives to reuse and to discard in household trash. International guidelines already emphasize that disposal strategies must be designed for the realities of home injection and must be coupled with education to achieve durable behavior change (8, 46–49).

This study’s strengths include its large, diverse sample from urban, peri-urban, and rural settings; standardized definitions for safe vs. unsafe disposal; clear distinction between pen needle and syringe behaviors; and robust models adjusted for key socioeconomic, clinical, and access-related factors. However, several limitations warrant consideration. First, self-reported behaviors are prone to social-desirability bias (likely underestimating unsafe practices) and recall error, though 30-day windows with visual aids are intended to minimize the latter. Second, the cross-sectional design precludes definitive causal inference: observed associations may reflect unmeasured confounding, reverse causation, or shared underlying determinants rather than direct causal effects. For example, the association between limited container availability and unsafe disposal could be confounded by rural residence, healthcare system engagement, or socioeconomic position beyond measured covariates, and reported community-safety incidents may influence subsequent reporting of disposal behaviors (reverse causation). While biological plausibility, dose–response patterns, consistency with experimental literature, and temporal precedence of some exposures (e.g., counseling and diabetes duration) strengthen causal interpretation for select associations, intervention trials are necessary to establish causal effectiveness. Third, the single-center design may limit generalizability despite geographic diversity within the catchment area. Fourth, residual confounding by unmeasured factors (e.g., depression, diabetes distress, and social support networks) cannot be excluded. Future research should prioritize pragmatic trials that bundle education with disposal infrastructure and assess both clinical (lipohypertrophy, dose variability, and hypoglycemia) and community outcomes (household injuries and municipal sharps reports), as well as implementation studies that evaluate kiosk density, pharmacy participation, and reimbursement reforms.

## 5 Conclusion

This study underscores that injection safety is not solely a matter of individual behavior but a systems responsibility that spans clinical practice, supply chains, and municipal infrastructure. The observed patterns indicate that structural determinants—access to puncture-resistant containers, proximity and visibility of disposal pathways, and routine, high-quality counseling—shape sharps disposal and needle-reuse practices in ways that directly affect both households and the broader community. Accordingly, diabetes care should be reframed as an integrated continuum that couples Forum for Injection Technique and Therapy Expert Recommendations (FITTER)-concordant education with assured access to single-use supplies and convenient, clearly signposted take-back options (pharmacy, kiosk, or mail-back), anchored by reimbursement and procurement policies that remove perverse economic incentives. Health services and public-health authorities should co-design and evaluate scalable, equity-focused implementations, with ongoing monitoring of clinical and community-safety metrics. Embedding these elements into standard care would align therapeutic excellence with environmental stewardship and occupational protection, advancing patient welfare and community safety as coequal aims of high-quality diabetes care.

## Data Availability

The raw data supporting the conclusions of this article will be made available by the authors, without undue reservation.

## References

[1] Thompson BM Cook CB. Unsafe sharps disposal among insulin-using patients with diabetes mellitus: An emerging global crisis. J Diabetes Sci Technol. (2022) 16:1376–80. doi: 10.1177/19322968211059851, PMID: 34852676 PMC9631533

[2] Montoya JM Thompson BM Boyle ME Leighton ME Cook CB. Patterns of sharps handling and disposal among insulin-using patients with diabetes mellitus. J Diabetes Sci Technol. (2021) 15:60–6. doi: 10.1177/1932296819882926, PMID: 31640410 PMC7782998

[3] Gold K. Analysis: the impact of needle, syringe, and lancet disposal on the community. J Diabetes Sci Technol. (2011) 5:848–50. doi: 10.1177/193229681100500404, PMID: 21880224 PMC3192588

[4] Wu J Wang M Yan H. Status of waste disposal of sharps outside medical institutions for patients with diabetes: a systematic review. PLoS One. (2023) 18:e0288993. doi: 10.1371/journal.pone.0288993, PMID: 37976255 PMC10655971

[5] Frid AH Kreugel G Grassi G Halimi S Hicks D Hirsch LJ . New insulin delivery recommendations. Mayo Clin Proc. (2016) 91:1231–55. doi: 10.1016/j.mayocp.2016.06.010, PMID: 27594187

[6] Ishtiaq O Qadri AM Mehar S Gondal GM Iqbal T Ali S . Disposal of syringes, needles, and lancets used by diabetic patients in Pakistan. J Infect Public Health. (2012) 5:182–8. doi: 10.1016/j.jiph.2012.02.002, PMID: 22541266

[7] Zabaleta-Del-Olmo E Vlacho B Jodar-Fernández L Urpí-Fernández AM Lumillo-Gutiérrez I Agudo-Ugena J . Safety of the reuse of needles for subcutaneous insulin injection: a systematic review and meta-analysis. Int J Nurs Stud. (2016) 60:121–32. doi: 10.1016/j.ijnurstu.2016.04.010, PMID: 27297374

[8] Tian T Aaron RE Huang J Yeung AM Svensson J Gentile S . Lipohypertrophy and insulin: An update from the diabetes technology society. J Diabetes Sci Technol. (2023) 17:1711–21. doi: 10.1177/19322968231187661, PMID: 37555266 PMC10658672

[9] Berlanda G Telo GH Gossenheimer AN Auler A da Silva ES Rodrigues PG . Impact of syringe and needle reuse on the clinical outcomes of patients with type 2 diabetes: a 12-week randomized clinical trial. Diabetes Care. (2024) 47:2146–54. doi: 10.2337/dc24-0157, PMID: 39405489

[10] Mader JK Fornengo R Hassoun A Heinemann L Kulzer B Monica M . Relationship between Lipohypertrophy, glycemic control, and insulin dosing: a systematic Meta-analysis. Diabetes Technol Ther. (2024) 26:351–62. doi: 10.1089/dia.2023.0491, PMID: 38215209 PMC11058417

[11] Charbonneau MS Parsons KA Danckert DC Silveira K Shcherbakova N Capoccia KL. Sharps disposal practices among people with diabetes in a community care clinic. Diabetes Spectr. (2022) 35:476–83. doi: 10.2337/ds21-0106, PMID: 36561648 PMC9668714

[12] Silva GS Gotschall JW Hu F Whidden E Cunningham P Niculcea J . An educational intervention to reduce regulated medical waste: the inpatient medicine and outpatient dermatology settings. Int J Dermatol. (2025) 64:901–8. doi: 10.1111/ijd.17682, PMID: 39923199 PMC12008608

[13] Kinrys G Gold AK Worthington JJ Nierenberg AA. Medication disposal practices: increasing patient and clinician education on safe methods. J Int Med Res. (2018) 46:927–39. doi: 10.1177/0300060517738681, PMID: 29322845 PMC5972255

[14] Blenkharn JI Odd C. Sharps injuries in healthcare waste handlers. Ann Occup Hyg. (2008) 52:281–6. doi: 10.1093/annhyg/men010, PMID: 18448444

[15] Moore D. Needle stick injuries in the community. Paediatr Child Health. (2008) 13:205–10. doi: 10.1093/pch/pxy129. 19252702 PMC2529409

[16] Yosef T Asefa A Amsalu H Setegn Alie M Habte A Ashuro Z . Occupational exposure to needle stick and sharp injuries and Postexposure prophylaxis utilization among healthcare professionals in Southwest Ethiopia. Can J Infect Dis Med Microbiol. (2025) 2025:3792442. doi: 10.1155/cjid/3792442, PMID: 40226431 PMC11986913

[17] Padmanabhan KK Barik D. Health hazards of medical waste and its disposal In: Energy from toxic organic waste for heat and power generation (2019). 99–118. doi: 10.1016/B978-0-08-102528-4.00008-0

[18] Song Z Guo X Ji L Huang X Hirsch LJ Strauss KW. Insulin injection technique in China compared with the rest of the world. Diabetes Ther. (2018) 9:2357–68. doi: 10.1007/s13300-018-0525-y, PMID: 30377996 PMC6250623

[19] Ji J Lou Q. Insulin pen injection technique survey in patients with type 2 diabetes in mainland China in 2010. Curr Med Res Opin. (2014) 30:1087–93. doi: 10.1185/03007995.2014.895711, PMID: 24552616

[20] Ji L Chandran A Inocencio TJ Sun Z Li Q Qin G . The association between insurance coverage for insulin pen needles and healthcare resource utilization among insulin-dependent patients with diabetes in China. BMC Health Serv Res. (2018) 18:300. doi: 10.1186/s12913-018-3095-9, PMID: 29699587 PMC5921994

[21] Pawaskar MD Camacho FT Anderson RT Cobden D Joshi AV Balkrishnan R. Health care costs and medication adherence associated with initiation of insulin pen therapy in Medicaid-enrolled patients with type 2 diabetes: a retrospective database analysis. Clin Ther. (2007) 29:1294–305. doi: 10.1016/j.clinthera.2007.07.007, PMID: 18046929

[22] Abujbara M Khreisat EA Khader Y Ajlouni KM. Effect of insulin injection techniques on glycemic control among patients with diabetes. Int J Gen Med. (2022) 15:8593–602. doi: 10.2147/ijgm.s393597, PMID: 36545247 PMC9762765

[23] Hawa A Teng CL Devaraj NK Saadatun A Rawaida AL Chong FY . Prevalence and associated factors of lipohypertrophy in insulin-injected patients with diabetes in selected primary care clinics in peninsular Malaysian: a cross-sectional study. Malays Fam Physician. (2023) 18:37. doi: 10.51866/oa.100 37449277 PMC10337597

[24] Zan H Liu T Meng Z Wang J. Localization of the questionnaire about sharps disposal at home among diabetes based on knowledge, attitude, and practice theory, and a cross-sectional survey of current conditions. Front Public Health. (2024) 12:1355510. doi: 10.3389/fpubh.2024.1355510, PMID: 38864009 PMC11165081

[25] Moore DL. Needle stick injuries in the community. Paediatr Child Health. (2018) 23:532–8. doi: 10.1093/pch/pxy129, PMID: 30894792 PMC6241928

[26] Tu H Lu X Wang J Sheng Z Liu D Li J . At-home disposal practices of used insulin needles among patients with diabetes in China: a single-center, cross-sectional study. Front Public Health. (2022) 10:1027514. doi: 10.3389/fpubh.2022.1027514, PMID: 36568796 PMC9772984

[27] Akbari H Ghasemi F Akbari H Adibzadeh A. Predicting needlestick and sharps injuries and determining preventive strategies using a Bayesian network approach in Tehran, Iran. Epidemiol Health. (2018) 40:e2018042. doi: 10.4178/epih.e2018042, PMID: 30130955 PMC6232661

[28] Huang L Katsnelson S Yang J Argyrou C Charitou MM. Factors contributing to appropriate sharps disposal in the community among patients with diabetes. Diabetes Spectr. (2018) 31:155–8. doi: 10.2337/ds17-0033, PMID: 29773935 PMC5951232

[29] Chen L Xing Q Li J Zhou J Yuan Y Wan Y . Injection technique education in patients with diabetes injecting insulin into areas of lipohypertrophy: a randomized controlled trial. Diabetes Ther. (2021) 12:813–26. doi: 10.1007/s13300-021-01013-1, PMID: 33570716 PMC7947164

[30] Cobden D Lee WC Balu S Joshi AV Pashos CL. Health outcomes and economic impact of therapy conversion to a biphasic insulin analog pen among privately insured patients with type 2 diabetes mellitus. Pharmacotherapy. (2007) 27:948–62. doi: 10.1592/phco.27.7.948, PMID: 17594200

[31] Markkanen P Galligan C Laramie A Fisher J Sama S Quinn M. Understanding sharps injuries in home healthcare: the safe home care qualitative methods study to identify pathways for injury prevention. BMC Public Health. (2015) 15:359. doi: 10.1186/s12889-015-1673-x, PMID: 25885473 PMC4414288

[32] Brouillette NM Quinn MM Kriebel D. Risk of sharps injuries to home care nurses and aides: a systematic review and meta-analysis. J Occup Environ Med. (2017) 59:1072–7. doi: 10.1097/jom.0000000000001160, PMID: 28930800 PMC5671783

[33] Quinn MM Markkanen PK Galligan CJ Kriebel D Chalupka SM Kim H . Sharps injuries and other blood and body fluid exposures among home health care nurses and aides. Am J Public Health. (2009) 99 Suppl 3:S710–7. doi: 10.2105/ajph.2008.150169 19890177 PMC2774204

[34] Conover S Koo H Boynton-Jarrett R. Spatiotemporal trends in discarded needle reports near schools in Boston, Massachusetts, between 2016-2019. Am J Drug Alcohol Abuse. (2021) 47:737–45. doi: 10.1080/00952990.2021.1978473, PMID: 34783625

[35] Awan UA Bashir S Hassan U Khan SN Awan FM Jabbar A . HPV-driven breast carcinogenesis: associations with tumor severity, Ki67 expression and metastasis. Infect Agents Cancer. (2025) 20:55. doi: 10.1186/s13027-025-00668-w, PMID: 40804747 PMC12345114

[36] Spollett G Edelman SV Mehner P Walter C Penfornis A. Improvement of insulin injection technique: examination of current issues and recommendations. Diabetes Educ. (2016) 42:379–94. doi: 10.1177/0145721716648017, PMID: 27216036

[37] Silver B Ramaiya K Andrew SB Fredrick O Bajaj S Kalra S . EADSG guidelines: insulin therapy in diabetes. Diabetes Ther. (2018) 9:449–92. doi: 10.1007/s13300-018-0384-6, PMID: 29508275 PMC6104264

[38] Kim KJ Choi JH Kim KJ An JH Kim HY Kim SG . Determinants of long-term durable glycemic control in new-onset type 2 diabetes mellitus. Diabetes Metab J. (2017) 41:284–95. doi: 10.4093/dmj.2017.41.4.284, PMID: 28868826 PMC5583406

[39] Chon S Lee YJ Fraterrigo G Pozzilli P Choi MC Kwon MK . Evaluation of glycemic variability in well-controlled type 2 diabetes mellitus. Diabetes Technol Ther. (2013) 15:455–60. doi: 10.1089/dia.2012.0315, PMID: 23617251 PMC3671661

[40] Awan UA Guo X Khattak AA Hassan U Khan S. Economic crises and cancer care in Pakistan-timely action saves lives. Lancet. (2024) 403:613–4. doi: 10.1016/s0140-6736(23)01380-6, PMID: 38368005

[41] Qasim M Awan UA Afzal MS Saqib MAN Siddiqui S Ahmed H. Dataset of knowledge, attitude, practices and psychological implications of healthcare workers in Pakistan during COVID-19 pandemic. Data Brief. (2020) 32:106234. doi: 10.1016/j.dib.2020.106234, PMID: 32895632 PMC7462453

[42] Khan MZ Hussain M Khan AA Hassan U Akhter N Hameed M . Frequency of non-diabetic renal disease in type 2 diabetes mellitus patients undergoing renal biopsy. J Ayub Med Coll Abbottabad. (2021) 33:S757–62. 35077622

[43] Saqib MAN Siddiqui S Qasim M Jamil MA Rafique I Awan UA . Effect of COVID-19 lockdown on patients with chronic diseases. Diabetes Metab Syndr. (2020) 14:1621–3. doi: 10.1016/j.dsx.2020.08.028, PMID: 32889403 PMC7450263

[44] Hareem A Lee J Stupans I Park JS Wang K. Benefits and barriers associated with e-prescribing in community pharmacy - a systematic review. Explor Res Clin Soc Pharm. (2023) 12:100375. doi: 10.1016/j.rcsop.2023.100375, PMID: 38145236 PMC10746557

[45] Kroenert AC Bertsche T. Implementation, barriers, solving strategies and future perspectives of reimbursed community pharmacy services - a nationwide survey for community pharmacies in Germany. BMC Health Serv Res. (2024) 24:1463. doi: 10.1186/s12913-024-11745-y, PMID: 39587619 PMC11590365

[46] Hood KK Hilliard M Piatt G Ievers-Landis CE. Effective strategies for encouraging behavior change in people with diabetes. Diabetes Manag. (2015) 5:499–510. doi: 10.1111/dme.12738, PMID: 30100925 PMC6086609

[47] Awan UA Riasat S Naeem W Kamran S Khattak AA Khan S. Monkeypox: a new threat at our doorstep! J Infect. (2022) 85:e47–8. doi: 10.1016/j.jinf.2022.05.027, PMID: 35649489 PMC9534065

[48] Khurram M Irshad A Alamgir M Awan UA Syed A Sadia H. Epidemiological survey of the prevalence of HCV and HBV among the factory workers in the periphery of Lahore. BioSci Rev. (2021) 3:25–33. doi: 10.32350/BSR.0301.03

[49] Iftikhar N Khattak AA Awan UA Saeed A Ayaz H Khan HI . Seroepidemiology of human cytomegalovirus and human herpesvirus 6 in a cohort of healthy blood donors from Abbottabad, Pakistan. BioSci Rev. (2025) 7:21–31. doi: 10.32350/bsr.72.03

