# Supplementary material for: Access, awareness, and risk: drivers of unsafe sharps disposal and needle reuse in insulin-treated adults in China
Source: Front Public Health. 2025 Nov 13;13:1698224. doi: 10.3389/fpubh.2025.1698224 (PMC12657182; doi:10.3389/fpubh.2025.1698224)
Supplement: Supplementary file 1 [file Data_Sheet_1.docx]

SUPPLEMENTARY APPENDIX A

Complete Study Questionnaire (English Version)

**Study Title:** Access, Awareness, and Risk: Drivers of Unsafe Sharps Disposal and Needle Reuse in Insulin-Treated Adults in China

**Study Site:** The Central Hospital of Enshi Tujia and Miao Autonomous Prefecture, Enshi, Hubei, China

# INSTRUCTIONS FOR INTERVIEWERS

- Read all questions exactly as written
- Show response cards when indicated
- Record "Prefer not to answer" or "Not sure" when offered by participant
- Ensure privacy for sensitive questions (disposal practices, reuse behaviors)
- Use calendar aids for recall periods (30 days, 3 months, 12 months)
- Complete all screening questions before proceeding to main questionnaire

# SECTION A: SCREENING & ELIGIBILITY

*[INTERVIEWER: Complete these questions first to determine eligibility]*

**Q1. Are you 18 years of age or older?**

- Yes → Continue
- No → Thank participant; not eligible

**Q2. Have you self-injected insulin at home within the past 30 days?**

- Yes → Continue
- No → Thank participant; not eligible

**Q3. Have you been living at your current residence for at least the past 3 months?**

- Yes → Continue
- No → Thank participant; not eligible

*[If all answers are "Yes," participant is eligible. Proceed to informed consent and main questionnaire]*

# SECTION B: DEMOGRAPHICS

**Q4. What is your age?**

- <30 years
- 30–44 years
- 45–59 years
- ≥60 years
- Prefer not to answer

**Q5. What is your sex?**

- Female
- Male
- Intersex
- Prefer not to answer

**Q6. What is the highest level of education you have completed?**

- No formal schooling
- Primary school
- Secondary school (middle school/junior high)
- Higher secondary (high school/senior high)
- College/University (bachelor's degree)
- Graduate or higher (master's/doctoral degree)
- Prefer not to answer

**Q7. What is your current employment status?**

- Employed (full-time)
- Employed (part-time)
- Self-employed
- Unemployed
- Student
- Homemaker
- Retired
- Prefer not to answer

**Q8. Which category best describes your total household income level?**

- Low
- Lower-middle
- Upper-middle
- High
- Prefer not to answer

# COMPLETE QUESTION LIST

| **Q#** | **Question Text** |
| --- | --- |
| **SECTION A: SCREENING** | |
| **Q1** | Are you 18 years of age or older? |
| **Q2** | Have you self-injected insulin at home within the past 30 days? |
| **Q3** | Have you been living at your current residence for at least the past 3 months? |
| **SECTION B: DEMOGRAPHICS** | |
| **Q4** | What is your age? |
| **Q5** | What is your sex? |
| **Q6** | What is the highest level of education you have completed? |
| **Q7** | What is your current employment status? |
| **Q8** | Which category best describes your total household income level? |
| **SECTION C: RESIDENCE & HOUSEHOLD** | |
| **Q9** | What type of area do you currently live in? |
| **Q10** | What is your housing status? |
| **Q11** | Are there any children under 12 years of age living in your household? |
| **Q12** | Do any household pets have access to areas where you dispose of household waste? |
| **SECTION D: CLINICAL CHARACTERISTICS** | |
| **Q13** | What type of diabetes have you been diagnosed with? |
| **Q14** | How long have you been diagnosed with diabetes? |
| **Q15** | What is your current insulin regimen? |
| **Q16** | What type of device do you primarily use to inject insulin? |
| **Q17** | When was your most recent hemoglobin A1c (HbA1c) blood test? |
| **Q18** | In the past 30 days, how often have you experienced vision problems or hand/finger difficulties that made it hard to handle your insulin injections or sharps safely? |
| **SECTION E: SHARPS DISPOSAL (30-day recall)** | |
| **Q19** | Thinking about the LAST time you disposed of used needles or sharps, where did you dispose of them? |
| **Q20** | In the past 30 days, how did you USUALLY dispose of your used needles, lancets, or other sharps? |
| **Q21** | At your LAST disposal, did you use a puncture-resistant container? |
| **Q22** | In the past 30 days, how often did you seal or close your sharps container after adding used needles? |
| **Q23** | In the past 30 days, did you label your sharps container (e.g., with "BIOHAZARD" or "USED NEEDLES")? |
| **Q24** | In the past 30 days, where did you typically store your sharps container or used needles at home? |
| **Q25** | In the past 30 days, how long did you typically wait before disposing of used needles in a container or trash? |
| **Q26** | In the past 30 days, how often did you recap needles before disposing of them? |
| **Q27** | In the past 30 days, how often did you bend or break needles before disposing of them? |
| **SECTION F: NEEDLE REUSE (30-day recall)** | |
| **Q28** | In the past 30 days, did you ever use the same needle for more than one insulin injection? |
| **Q29** | In the past 30 days, on average, how many separate injections did you perform with the same needle? |
| **Q30** | In the past 30 days, what is the MAXIMUM number of times you used a single needle? |
| **Q31** | What were your main reasons for reusing needles? [SELECT ALL THAT APPLY] |
| **SECTION G: SUPPLY AVAILABILITY** | |
| **Q32** | Thinking about your insulin injection supplies at home or at places where you inject, how would you describe the availability of sterile, unused needles? |
| **Q33** | In the past 30 days, on how many days did you NOT have sufficient sterile (unused) needles available? |
| **SECTION H: COMMUNITY SAFETY** | |
| **Q34** | In the past 12 months, has anyone in your household accidentally been stuck or pricked by one of your used insulin needles? |
| **Q35** | "It is safe to dispose of used insulin needles in regular household trash as long as the needle is recapped." Do you agree or disagree with this statement? |
| **Q39** | In the past 3 months, have you personally seen loose (uncovered) insulin needles or syringes in community areas such as parks, sidewalks, or public waste areas? |
| **Q40** | In the past 12 months, are you aware of any waste collection worker being injured by one of your discarded sharps? |
| **SECTION I: KNOWLEDGE & ACCESS** | |
| **Q36** | In the past 12 months, have you received counseling or instruction from a healthcare professional (doctor, nurse, diabetes educator, pharmacist) on how to safely dispose of used insulin needles and sharps? |
| **Q37** | Are you aware of any pharmacy or clinic take-back program where you can return used insulin needles and sharps for safe disposal? |
| **Q38** | Approximately how long does it take you to travel from your home to the nearest location where you can safely dispose of used needles (e.g., pharmacy drop-off, clinic, approved disposal site)? |

CLOSING

**Thank you for participating in this important study. Your responses will help improve diabetes care and community safety.**

***[INTERVIEWER: Provide participant with:]***

- Information sheet on safe sharps disposal
- Local pharmacy/clinic take-back program contacts
- Free puncture-resistant container (if available)
- Contact information for study team if questions arise

___________________________________________

Corresponding Author: Fang Wang, wff_es@163.com

Ethics Approval: The Central Hospital of Enshi Tujia and Miao Autonomous Prefecture IRB
